# Case Report: Conversion therapy with chemotherapy, antiangiogenic therapy, and immunotherapy for locally advanced synchronous gastric hepatoid adenocarcinoma and poorly differentiated adenocarcinoma

**DOI:** 10.3389/fonc.2026.1898989

**Published:** 2026-09-25

**Authors:** Yin Shu, Feiyang Yan, Paerhati Shayimu, Weina Wang, Yajie Chen, Wenbin Zhang

**Affiliations:** 1 The Third Clinical Medical College of Xinjiang Medical University, Urumqi, Xinjiang, China; 2 Department of Gastrointestinal Surgery II, Affiliated Tumor Hospital of Xinjiang Medical University, Urumqi, Xinjiang, China; 3 Department of Pathological Diagnosis, Affiliated Tumor Hospital of Xinjiang Medical University, Urumqi, Xinjiang, China

**Keywords:** conversion therapy, gastric cancer, hepatoid adenocarcinoma of the stomach, immunotherapy, multidisciplinary team

## Abstract

Hepatoid adenocarcinoma of the stomach (HAS) is a rare, aggressive gastric carcinoma that is frequently associated with elevated alpha-fetoprotein (AFP). We report a patient with resolved hepatitis B virus infection and two spatially distinct gastric lesions: poorly differentiated adenocarcinoma in the gastric body and HAS in the gastric angularis. The disease was initially considered unresectable. After multidisciplinary evaluation, the patient received oxaliplatin, S-1, sintilimab, and apatinib. Treatment produced radiological regression and AFP normalization, enabling total gastrectomy. Histopathology showed no viable carcinoma in either tumor bed or in 20 regional lymph nodes, confirming a pathological complete response. This case illustrates the potential role of individualized conversion therapy in selected patients with alpha-fetoprotein-producing gastric cancer. It does not establish the four-agent regimen as standard care. Interpretation is limited by the single-patient design, short follow-up, absence of lesion-specific sequencing and serial hepatitis B virus DNA data, and a nonstandardized maintenance strategy.

## 1 Introduction

Gastric cancer remains a major global health burden and a leading cause of cancer-related mortality worldwide (1). Alpha-fetoprotein-producing gastric cancer (AFPGC) and hepatoid adenocarcinoma of the stomach (HAS) are uncommon, aggressive entities with poor outcomes (2, 3). AFPGC is defined by AFP production, whereas HAS is a histological diagnosis based on hepatoid differentiation. The categories overlap but are not interchangeable. AFPGC frequently exhibits vascular invasion, early liver metastasis, and resistance to conventional therapy (2, 4).

AFPGC and HAS usually present with nonspecific symptoms and are often diagnosed at an advanced stage. The differential diagnosis is more difficult when synchronous gastric tumors have distinct histological phenotypes. Diagnosis requires integration of gastroscopy, cross-sectional imaging, and pathological assessment. AFP, glypican-3, and SALL4 can support hepatoid differentiation, whereas the Ki-67 index, human epidermal growth factor receptor 2 status, and programmed death-ligand 1 expression may inform treatment planning (2, 3). Multifocal disease with histological heterogeneity therefore benefits from multidisciplinary team evaluation (5, 11).

We report a patient with resolved hepatitis B virus (HBV) infection and synchronous gastric body and angularis tumors with different histological phenotypes (6, 7). Pathology showed poorly differentiated adenocarcinoma in the gastric body and HAS in the gastric angularis. The disease was initially considered unresectable because of serosal invasion and regional lymph-node involvement. Conversion therapy with oxaliplatin, S-1, sintilimab, and apatinib produced tumor regression and AFP normalization (8). Radical resection subsequently confirmed a pathological complete response (pCR), an outcome rarely reported in HAS (9, 10).

This case addresses the diagnostic and therapeutic uncertainty created by synchronous gastric tumors with divergent histology and marked AFP elevation. It also provides a clinically relevant example of conversion to curative-intent resection after multidisciplinary treatment. Because the evidence derives from one patient, the regimen should be regarded as hypothesis-generating rather than practice-defining.

## 2 Case presentation

### 2.1 Patient information

A 60-year-old man had a 30-year history of HBV infection without previous antiviral treatment. Serology was positive for hepatitis B e antibody (HBeAb), hepatitis B core antibody (HBcAb), and hepatitis B surface antibody (HBsAb). In March 2025, he presented with a 4-month history of back pain, and subsequent evaluation revealed a gastric lesion. His Eastern Cooperative Oncology Group performance status (ECOG PS) was 1, Nutrition Risk Screening 2002 (NRS-2002) score was 4, and numeric rating scale pain score was 3. Physical examination showed mild epigastric tenderness without other notable findings.

### 2.2 Clinical examinations

Gastroscopy on March 6, 2025, identified malignant lesions in the gastric body and gastric angularis (**Figure 1**). Normal mucosa separated the lesions, with no continuous infiltration between them. Abdominal computed tomography (CT) showed wall thickening along the lesser curvature and antrum, transmural serosal invasion, an antral ulcer, and multiple enlarged perigastric lymph nodes suspicious for metastases. The tumor also involved the left gastric artery. Serum AFP was 1,851.77 ng/mL, and carcinoembryonic antigen was 5.85 µg/L.

**Figure 1:**
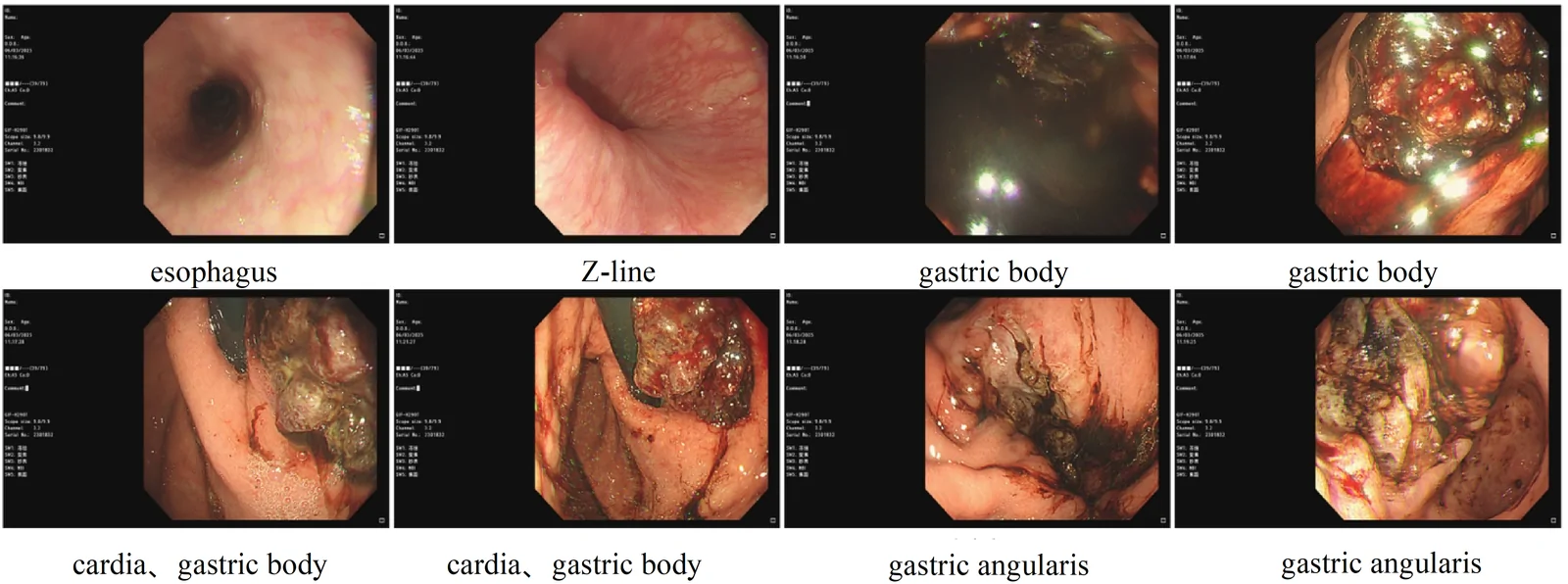
Pretreatment gastroscopy of the spatially separate gastric lesions. The upper row shows the esophagus, Z-line, and two views of the gastric body lesion. The lower row shows two views of the cardia/gastric body and two views of the gastric angularis lesion. Normal mucosa separated the two malignant lesions.

Biopsy confirmed two spatially distinct malignancies: HAS in the gastric angularis and poorly differentiated adenocarcinoma in the gastric body. The diagnosis of synchronous primary gastric cancers was based on separate endoscopic locations, independent biopsies, and divergent histology rather than AFP elevation alone. Immunohistochemistry showed p53 overexpression (95%, consistent with a missense pattern), retained MLH1, MSH2, MSH6, and PMS2, a Ki-67 index of 80%, E-cadherin positivity, HER2 score 0, and positivity for AE1/AE3 and BRG1. The angularis lesion showed focal AFP expression and was positive for glypican-3 and SALL4; INSM1 and synaptophysin were negative (**Figure 2**). Epstein-Barr virus-encoded small RNA *in situ* hybridization was negative. Programmed death-ligand 1 testing with the DAKO 22C3 antibody yielded a combined positive score of 2. Lesion-specific genomic sequencing was not performed, so molecular concordance between the tumors and its relationship to treatment response remain unknown.

**Figure 2:**
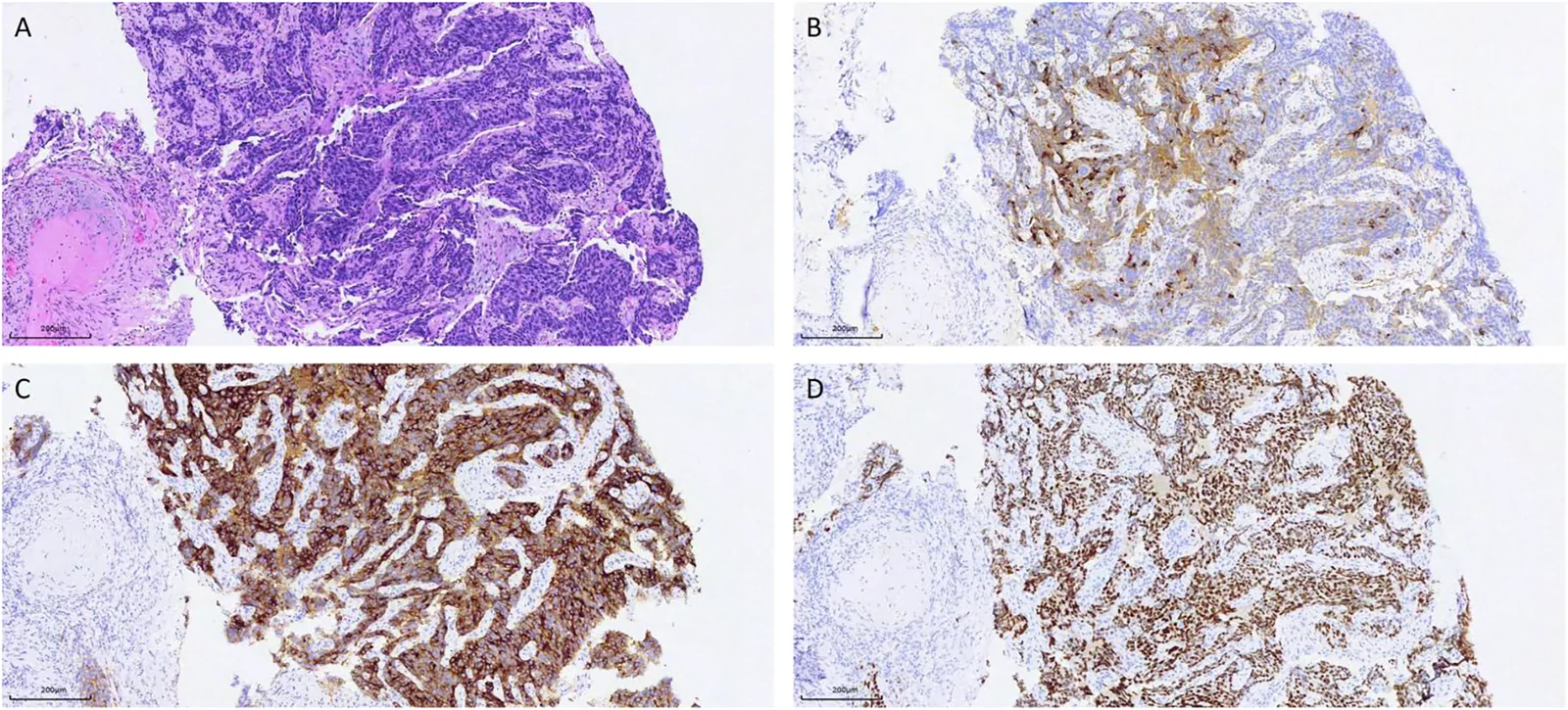
Pretreatment histopathology of the gastric angularis HAS. **(A)** Hematoxylin and eosin staining. **(B)** Focal AFP expression. **(C)** Glypican-3 expression. **(D)** SALL4 expression. Immunohistochemistry in panels B-D was performed using the EnVision method. Scale bars, 200 µm.

### 2.3 Treatment course

Following the 2025 Chinese Society of Clinical Oncology Guidelines for Gastric Cancer, the multidisciplinary team selected conversion therapy for unresectable, HER2-negative, mismatch repair-proficient/microsatellite-stable AFPGC. The four-agent regimen comprised oxaliplatin, S-1, sintilimab, and apatinib. A low programmed death-ligand 1 combined positive score was not considered sufficient to exclude immunotherapy in this high-risk setting. Before treatment, hepatitis B surface antigen and hepatitis B e antigen were negative, whereas hepatitis B surface, e, and core antibodies were positive. Serum HBV DNA was below the lower limit of quantification, consistent with resolved infection and no evidence of active replication at baseline. Four cycles were administered from March 11 to June 1, 2025. Back pain resolved after the first cycle, oral intake normalized after the second, and mild thrombocytopenia developed during the third. After four cycles, serum AFP decreased from 1,851.77 to 2.12 ng/mL, and CT showed marked tumor regression. The best radiological response was partial response (**Figure 3**). During treatment, transaminase levels were monitored, but complete longitudinal numerical results were not available for this report. Serial HBV DNA measurements were not available.

**Figure 3:**
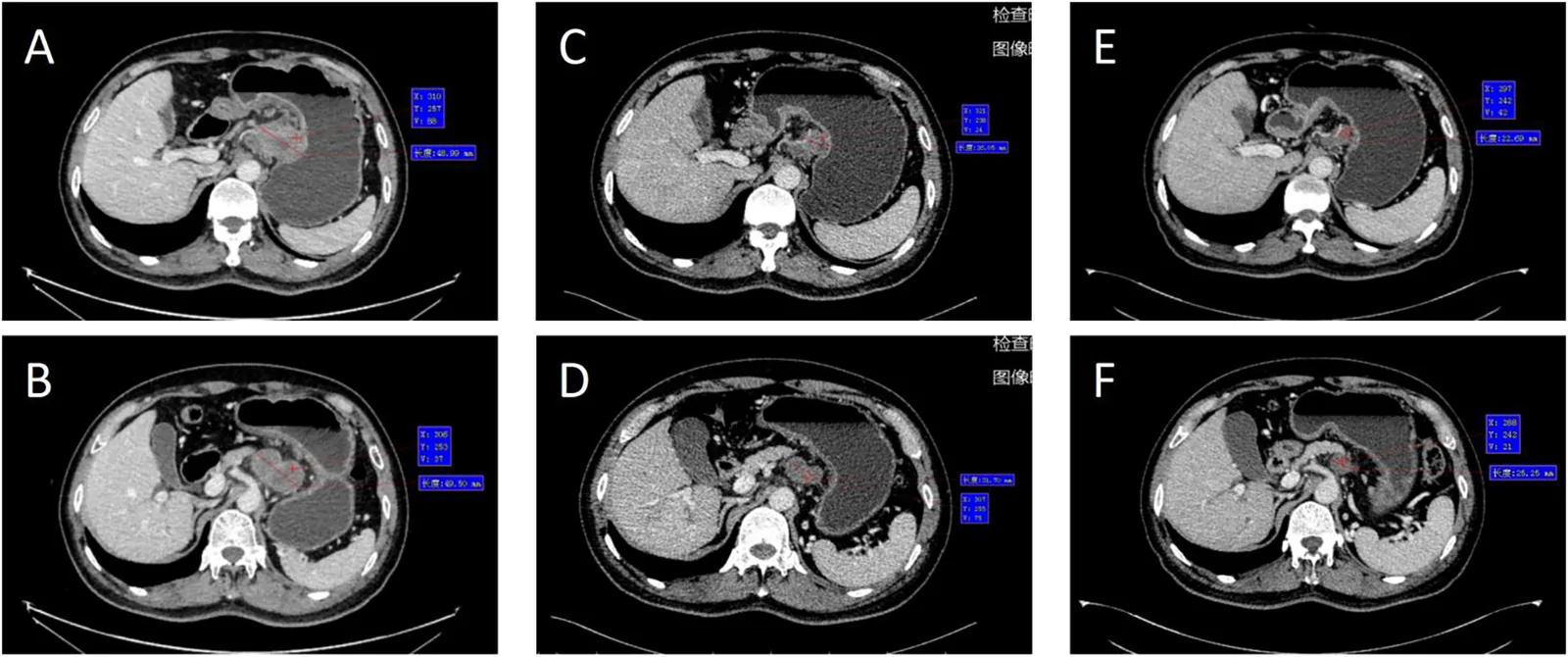
Serial contrast-enhanced CT images during conversion therapy. Red arrows indicate the primary gastric lesion. **(A, B)** Baseline images obtained on March 6, 2025. **(C, D)** Images obtained after two cycles of conversion therapy. **(E, F)** Images obtained after four cycles of conversion therapy.

The patient underwent laparoscopic total gastrectomy with curative intent on July 8, 2025. Preoperative nutritional status improved, with a 2-kg weight gain. Exploration showed no peritoneal dissemination, and scar-like changes at the lesser curvature were consistent with treatment response (**Figure 4**). Histopathology showed no viable carcinoma in either tumor bed; only fibrosis and inflammatory infiltration remained. Resection margins were negative, and no lymphovascular or perineural invasion was identified. All 20 regional lymph nodes were tumor-free, although six showed treatment-related regression. Tumor regression grade was 0, confirming a pCR (**Figure 5**).

**Figure 4:**
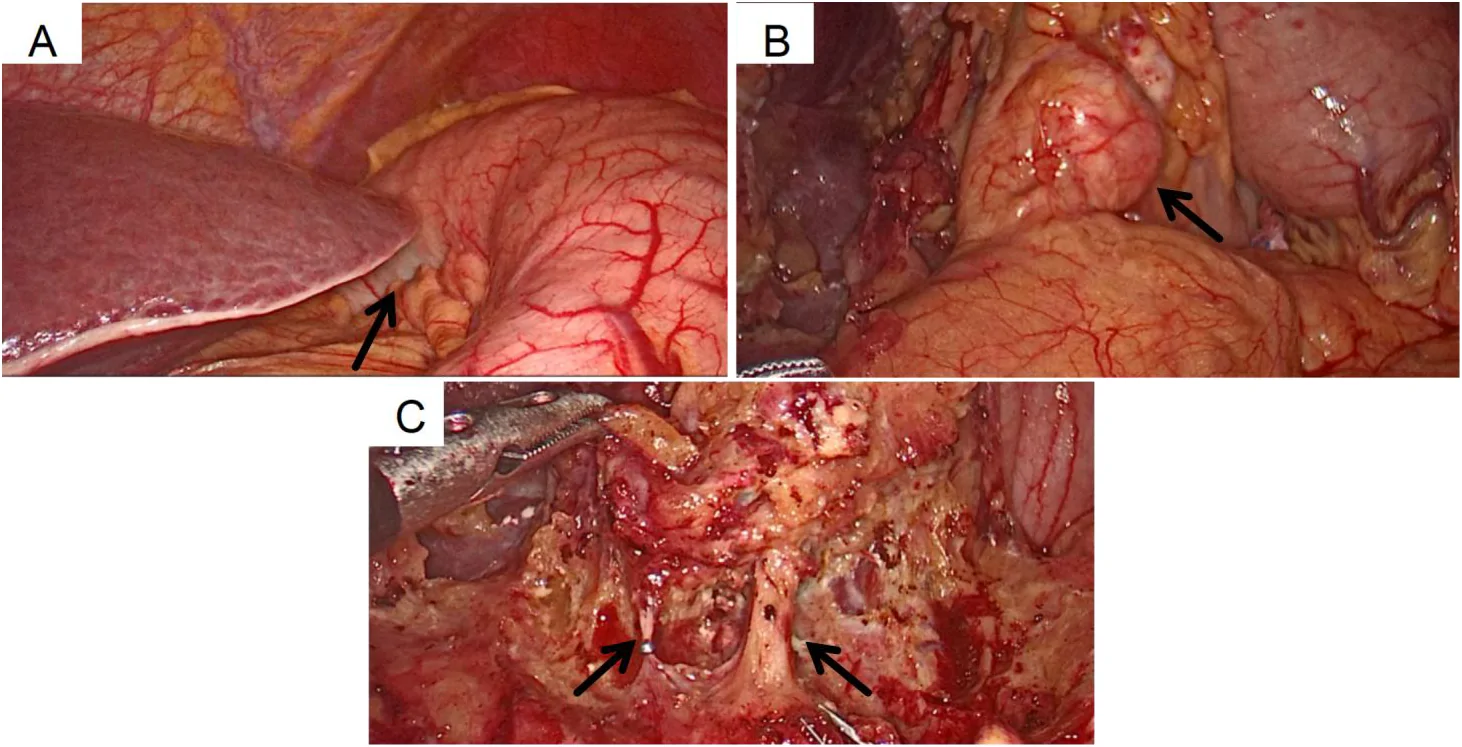
Intraoperative findings after conversion therapy. **(A)** Abdominal exploration showing no visible peritoneal dissemination. **(B)** Lymph nodes encasing the left gastric artery. **(C)** The coronary vein and left gastric artery after lymphadenectomy.

**Figure 5:**
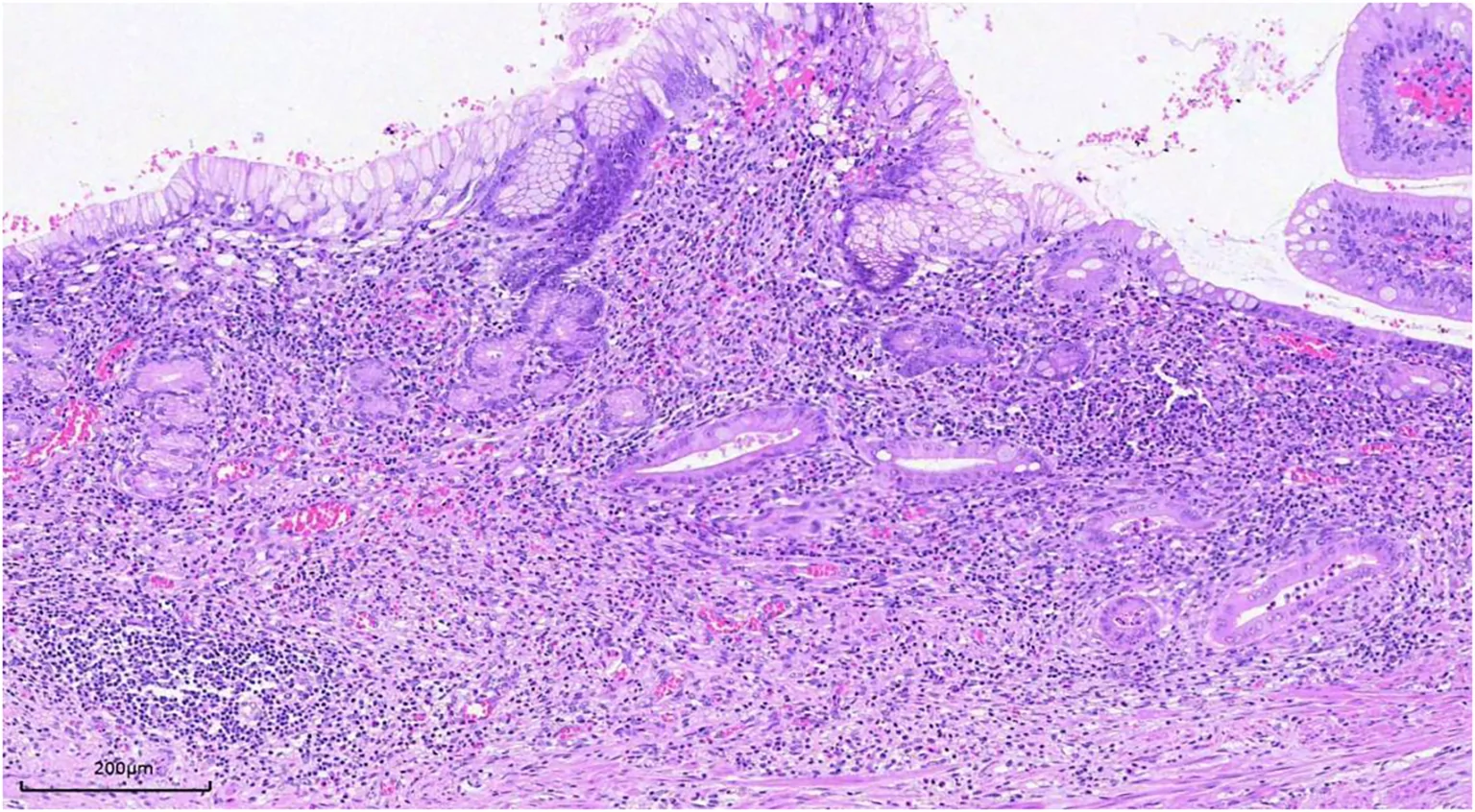
Post-treatment gastric tumor bed showing fibrosis and inflammatory infiltration without viable carcinoma (hematoxylin and eosin staining; scale bar, 200 µm).

Postoperative recovery was uneventful. Ambulation began at 18 hours, and the gastric tube was removed at 20 hours. Bowel function resumed on postoperative day 2, a full liquid diet began on day 3, and the abdominal drain was removed on day 6. The patient was discharged on day 8. At the 2-week review, the incision had healed and the patient was clinically well.

The same four-agent regimen was resumed on August 6, 2025, and two postoperative cycles were completed by September 18. Serum AFP and other gastric cancer-associated tumor markers remained within normal ranges (**Figure 6**). This maintenance treatment regimen was individually tailored and jointly developed by our center’s multidisciplinary team based on single-center experience, and the timing of treatment discontinuation was determined accordingly.

**Figure 6:**
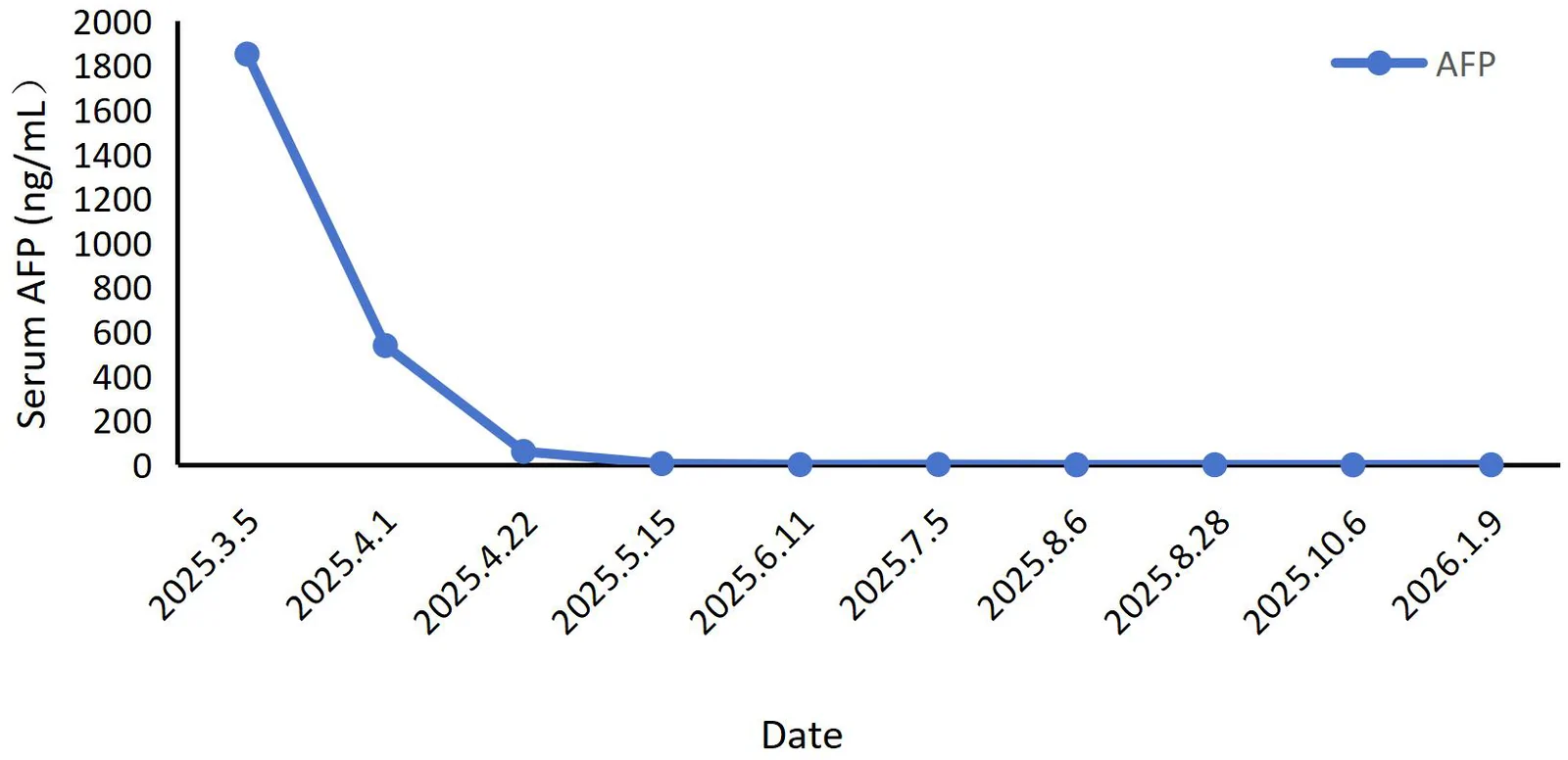
Serial serum AFP levels during conversion therapy, radical surgery, and postoperative treatment. AFP decreased from 1,851.77 ng/mL at baseline to within the reference range after conversion therapy and remained within that range during the available follow-up.

### 2.4 Follow-up and prognosis

CT examinations on October 15, 2025, and January 9, 2026, showed no recurrence. Upper gastrointestinal endoscopy on February 4, 2026, showed no anastomotic abnormality. The available follow-up was short and does not establish durable disease control.

### 2.5 Literature review

AFPGC and HAS overlap clinically but represent different diagnostic concepts. AFPGC is defined by AFP production, whereas HAS requires hepatoid morphology; either feature may occur without the other (2, 3, 12–14). Both entities are associated with vascular invasion, early liver metastasis, and poor prognosis (2, 17). Marked AFP elevation can also mimic hepatocellular carcinoma, particularly in patients with prior liver disease (2, 12). Synchronous gastric tumors with divergent histology are uncommon and require separate sampling to distinguish independent primaries from continuous or metastatic disease (5, 15).

Evidence for multimodal conversion therapy in AFPGC remains limited. The CAP 06 phase II trial reported a 66.7% confirmed objective response rate with camrelizumab, apatinib, and S-1 plus oxaliplatin in AFP-producing gastric or gastroesophageal junction adenocarcinoma (8). Other phase II studies support combining chemotherapy with immune-checkpoint or antiangiogenic therapy in selected gastric cancers, but they do not establish a HAS-specific standard (19, 20). Reviews of molecularly defined gastric cancer further emphasize that immunotherapy decisions depend on biological context rather than one marker alone (16). Preclinical studies suggest that oxaliplatin-induced immunogenic cell death and apatinib-mediated vascular normalization may enhance antitumor immunity (18, 21, 22). These data provide biological plausibility, not proof that each component contributed equally in this patient.

## 3 Discussion

The spatial separation and divergent histology of the gastric body and angularis lesions supported synchronous primary tumors. This distinction was clinically relevant because the lesions could differ in AFP production, molecular features, and treatment sensitivity. However, lesion-specific sequencing was not performed. Molecular concordance, clonal relatedness, and lesion-level predictors of response therefore remain unresolved.

The marked response is notable because the tumors were HER2-negative, mismatch repair-proficient/microsatellite-stable, and had low programmed death-ligand 1 expression. Nevertheless, a single response cannot identify the active component of a four-agent regimen or determine whether the same approach would benefit other patients. The result should therefore be interpreted as hypothesis-generating evidence for multidisciplinary conversion therapy in a carefully selected patient, not as support for universal standard care.

Three practical lessons emerge. First, elevated AFP in a patient with prior HBV infection should not be attributed to hepatocellular carcinoma without adequate imaging and pathological assessment. Second, spatially separate gastric tumors should be biopsied independently because histological heterogeneity can alter diagnosis and treatment selection. Third, multidisciplinary review can integrate pathology, biomarkers, serial AFP, imaging, and surgical assessment. These principles may be particularly relevant in HBV-endemic regions, where AFP interpretation is challenging.

Baseline serology and an HBV DNA level below the lower limit of quantification supported resolved HBV infection, but they did not replace longitudinal virological surveillance. Although liver enzymes were monitored during treatment, complete longitudinal numerical results were not available for this report. Serial HBV DNA measurements were also unavailable. These data limitations prevent a full assessment of treatment-related HBV reactivation. Future use of immune-checkpoint blockade in similar patients should include baseline and serial HBV DNA, alanine aminotransferase, and aspartate aminotransferase measurements, with antiviral management guided by hepatology assessment.

The postoperative maintenance strategy also requires cautious interpretation. Two additional cycles of the same four-agent regimen were administered, but the optimal regimen, number of cycles, duration, and stopping criteria are unknown. The absence of a comparator prevents assessment of whether maintenance therapy contributed to the observed short-term disease control. Follow-up should include symptoms, nutritional status, serial AFP and other tumor markers, cross-sectional imaging, endoscopy when indicated, and monitoring for delayed immune-related and hepatic adverse events.

This report has several important limitations. It is based on a single patient, involves a short follow-up period, and lacks a control group. Lesion-specific sequencing and paired pre- and post-treatment analyses were not performed, leaving inter-lesion molecular and immune heterogeneity unresolved. In addition, serial HBV DNA measurements and complete longitudinal numerical liver-enzyme results were unavailable, and the maintenance treatment strategy was neither predefined nor standardized. These limitations preclude any causal attribution to individual treatment components and limit the generalizability of the four-agent regimen. Future prospective multicenter studies should include lesion-specific molecular profiling, standardized radiological assessments, predefined treatment and maintenance protocols, longitudinal HBV monitoring, and comparative outcome evaluation.
